# Dental Whitening Gels: Strengths and Weaknesses of an Increasingly Used Method

**DOI:** 10.3390/gels5030035

**Published:** 2019-07-04

**Authors:** Luca Fiorillo, Luigi Laino, Rosa De Stefano, Cesare D’Amico, Salvatore Bocchieri, Giulia Amoroso, Gaetano Isola, Gabriele Cervino

**Affiliations:** 1 Department of Biomedical and Dental Sciences and Morphological and Functional Imaging, School of Dentistry, Messina University, 98100 Messina, Italy; 2 Department of Biomedical and Surgical and Biomedical Sciences, Naples University, 80100 Naples, Italy; 3 Department of Biomedical and Dental Sciences and Morphological and Functional Imaging, Messina University, 98100 Messina, Italy; 4 Department of General Surgery and Surgical-Medical Specialties, School of Dentistry, University of Catania, 95124 Catania, Italy

**Keywords:** tooth bleaching, bleaching agents, tooth bleaching agents, dentin sensitivity, esthetics, dental

## Abstract

Many people nowadays undergo treatments to improve their aesthetics, often neglecting the general state of health. Aesthetics and appearance have become of prime importance, perhaps correlating with of the advent of social networks and digital photographs. One of the most requested aesthetic treatments for dentists is dental bleaching through the use of whitening gels. Dental bleaching is a treatment which involves an improvement in the chrome of the teeth in a short time, and this treatment appears not invasive for the patients. In-office and at-home bleaching treatments can be found. The purpose of this scientific study is to evaluate all of the advantages and disadvantages of this medical treatment. In this study, were report information and items related to bleaching side effects. Dentists often find themselves in disagreement on this topic. The PICO (Population/Intervention/Comparison/Outcome) question investigated was: Are dental patients who have dental bleaching an increased risk of teeth damage? All of the data in the literature has been collected, and all of the side effects of this treatment were evaluated. 263 studies emerged from initial research; only 14 were screened after screening, as they contained sufficient data to evaluate the side effects of treatment. One certain thing emerged; among the contraindications to the treatment of dental bleaching dentinal hypersensitivity could be cited. In fact, one of the most reported undesirable effects regards this problem. Other studies have evaluated pain, color improvement and duration, or patient satisfaction. Dental bleaching is a treatment that aims to improve the aesthetics of our patients; this is also reflected from a psychological point of view and therefore has effects on general health. However, the whitening treatment is not suitable for everyone and must be carefully evaluated by a specialist. This treatment also entails maintenance by the patient and therefore better compliance to normal oral hygiene procedures.

## 1. Introduction

Despite being a particularly resistant and mineralized material, enamel retains a certain porosity and as such can be traversed by substances and particles capable of changing the color of the dentin, and therefore of the enamel itself. Insufficient oral hygiene favors the deposit of plaque, and therefore of tartar. The yellowish color and the barrier effect of the plaque that is opposed to the reflection of light gradually extinguish the brightness of the teeth with gradual color change. The colored particles of food, beverages and tobacco adhere tenaciously to mature plaque and tartar on enamel. The result is that the teeth appear dark, yellowed, opaque and less bright. After a deposit of 12 h of plaque, this oxidizes by evolving into tartar, and at this point only professional hygiene can remove it. Following scaling, a bleaching treatment can be performed. As the teeth progress with age, the tooth enamel becomes thinner due to wear and this allows the underlying dentin to become more visible [1,2]. This phenomenon involves a yellowing effect depending on the intrinsic color of the dentin; moreover, the permeability of the teeth allows the penetration of significant organic pigments (from chromogenic substances present in food, in drinks, in tobacco) that can cause a yellowing effect. In addition, trauma can cause calcific metamorphosis (calcification of the pulp chamber and/or of the root canal or of both), being able to produce a noticeable yellowing of the tooth which is difficult to treat. Many discolorings can be corrected, or considerably improved, through conservative methods such as bleaching, micro abrasion or macro abrasion, or the application of dental veeners. The slight discolorations are left intact and not be subjected to bleaching or to conservative treatment with micro abrasion or macro abrasion. Bleaching refers to the improvement of chromatic and aesthetic characteristics of a tooth through the application of a chemical agent to oxidize the organic pigmentation. Whitening techniques can be classified according to the vitality of the teeth and according to the place of execution of the procedure (in the outpatient department or externally). It is believed that the mechanism of action of teeth whitening with hydrogen peroxide gel is the oxidation of organic pigments, even if the chemical process is not yet well understood. Bleaching, in general, has an approximate duration ranging from one to 3 years. In all bleaching techniques there is a temporary decrease in the potential bonding strength of the composite when it is applied to the whitened and etched enamel [2,3]. This reduction in bond strength is caused by the residual oxygen or peroxide remaining in the tooth, which prevents the adhesive resin from being taken, which in turn precludes the formation of appropriate resin sapphires in the etched enamel. However, no loss of bond strength is observed if the restoration treatment with the composite is postponed for at least a week after discontinuing any bleaching [4]. In some cases the endodontically treated dental elements, especially if not correctly performed, can go against tooth discolouration and therefore worsen the aesthetic characteristics. These could require dental bleaching. Non-vital teeth bleaching procedures include an outpatient thermocatalytic technique and a technique outside the ambulatory called walking bleach (walking whitening). Non-vital teeth bleaching is quite effective, but involves a slight risk (1%) of causing a deleterious side effect, cervical reabsorption. To reduce the risk of reabsorption, a paste based on calcium hydroxide powder and sterile water in the pulp chamber is applied immediately after bleaching; furthermore, sodium perborate alone can be used as the main bleaching agent rather than in combination with hydrogen peroxide [2,3,4,5,6,7,8]. Generally, the indications for the various life whitening techniques are similar; these concern teeth with intrinsic discoloration due to aging, trauma or ingestion of drugs. The vital whitening procedures include an outpatient technique called power bleaching and an alternative technique outside the clinic prescribed by the dentist and performed at home by the patient (the so-called vital whitening with shower or night plate). The vital whitening technique in the clinic has the advantages of being completely under the dentist’s control, the soft tissue is generally protected and is able to whiten the teeth more quickly. The disadvantages mainly refer to the cost and duration of the treatment. The advantages of the home care technique are the use of a lower peroxide concentration, simple application, minimal side effects, lower cost for the shortest time to the chair required for treatment [2]. The disadvantages concern the risk of changes in the soft tissue for use which is too extensive [1,5]. The aim of the study is to evaluate this now-widespread dental practice, which is often confused with a purely aesthetic and non-medical treatment, and to evaluate all the contraindications and side effects it may have.

## 2. Results

### 2.1. Study Selection

**Figure 1:**
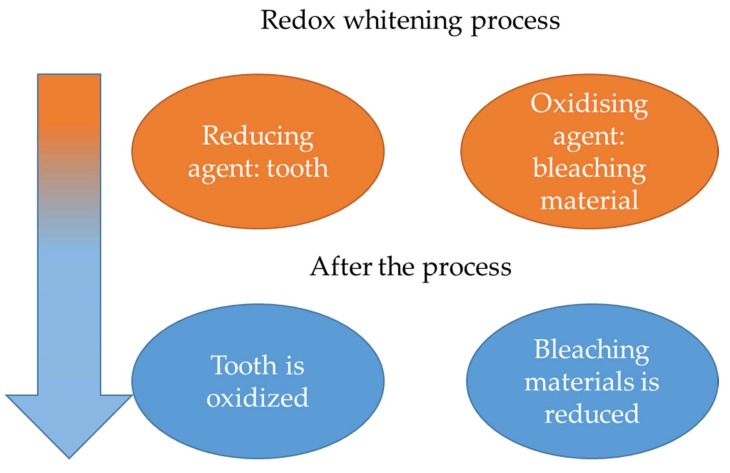
Redox whitening process.

After the research according to Section 5.2, the scientific search engines produced 263 results. At this point, results older than 10 years were eliminated (according to Figure 1), thus obtaining 121 results. All studies not containing information on the topic of the review, on the subject treated or on the side effects were removed from the results, obtaining 115 results. At this point other results were eliminated as they were not randomized controlled trials (RCTs) or clinical trials (CTs), and the full texts were eliminated. The result after an individual screening by the authors was 14 studies were included [9]. 

### 2.2. Study Characteristics

The results of the individual articles in the research were carefully evaluated. The results that can be obtained through the analysis of the literature are quite clear; the dental whitening treatment causes dentinal hypersensitivity, which can be reversible. This treatment should be carefully evaluated by the clinician, who should not perform it on patients who already suffer from dentinal hypersensitivity. However, it must be added that the tests evaluated on the quality of life related to oral health were been positive, so there is a positive psychological component that should not be underestimated (Table 1). 

### 2.3. Synthesis of Results

The summary results are shown in Table 1, a list of side effects has been summarized below:

- Tooth sensitivity
- Skin cold sensation
- Pain
- Difficulty in oral hygiene

According to the studies, these negative side effects seem to be transient; other values have been evaluated as an index related to the quality of life (QoL) or the visual analogue scale (VAS; see Appendix A). 

## 3. Discussion

### 3.1. Type of Dental Bleaching

**Figure 2:**
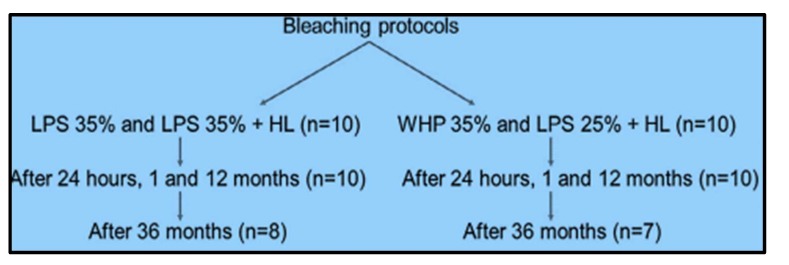
Mondelli et al. bleaching gels protocols.

Dental bleaching is a frequently-requested aesthetic treatment; as such it has now assumed considerable importance both in the domestic and in the professional field. In fact, white and wholesome teeth represent one of the most desired and sought-after aesthetic features. Under this term, generically, we group any treatment that leads the teeth to appear whiter. The fault, in many cases, is due to unfavorable genetic characteristics, to smoking, to the passage of time, or to the consumption of particular foods or drinks, such as coffee, licorice, tea, and artificial colors [5,6]. Teeth whitening is indicated to treat dental discolorations that may occur in the course of an individual’s life. In this regard, remember that such discolorations may be superficial (such as those caused by excessive consumption of coffee or tobacco), or more or less profound (such as those caused by the intake of some types of drugs). Furthermore, dental bleaching can be performed both on vital teeth and on teeth that have lost their vitality (devitalized teeth). In the latter case, however, the procedure is slightly more complex and requires longer lead times. There are different treatments to whiten one’s teeth. In general, however, the teeth can be made whiter in two ways: by rubbing (mechanical bleaching), or by bleaching substances (chemical bleaching). In any case—regardless of whether it is mechanical or chemical—teeth whitening procedures are substantially of two types: professional or at home. Professional treatment, of course, must be done by the dentist in specialized studios or medical aesthetic centers; while home treatment can be done at home by using certain products. Alternatively, you can whiten your teeth by performing combined treatments (dental practice, specialized center–home). Professional whitening, as mentioned, must be carried out in dental clinics—or at specialized centers—directly by the dentist or, possibly, by the dental hygienist. Professional whitening is bleaching carried out on the chair by the use of whitening chemical agents that can be activated, or not, by possible light sources. Naturally, the professional whitening results are more effective than the home procedures in terms of speed and degree of whitening obtained. Before proceeding with any whitening method, it is necessary to perform a thorough dental cleaning, in order to remove tartar, plaque and possible external pigmentations. Only after having performed the scaling is it possible to proceed with the actual whitening. Bleaching involves a chemical reaction of oxidation-reduction, in which the tooth goes against oxidation, also oxidizing all the pigments present on the enamel, and reducing the whitening material (Figure 2) [24,25,26].

The composition of the bleaching gels can be in an unexpanded, foamed or in a foamable state. The concentrations of the active ingredient, hydrogen peroxide (at different concentrations) or carbamide, do not vary. On the other hand, a foaming composition may include a higher concentration of peroxide in the unexpanded state so that with the foaming of the peroxide, the concentration may be the same or higher than the level present in a typical gel. For a non-foamed gel system, additional components may be needed to obtain a stable gel. These can include gelling agents, gel stabilizers, humectants and other adjuvants to improve the consistency of the gel, which can be added to one or both components, in the case of two-component whitening solutions. Gelling agents which can be used in the preparation of bleaching gels include, for example, cellulosic rubbers, fumed silica, for example, CAB-O-SIL^®^ pyrogenic silica provided by Cabot Corporation^®^, and emulsifying waxes such as Polawax^®^ (NF (National Formulary) emulsifying wax) or Crodafos CES^®^ (cetearyl alcohol (e) dicetyl phosphate (e) ceteth-10 phosphate), in their mixtures, in amounts to provide a stable gel. Some examples of cellulosic rubber include Klucel GF^®^, a hydroxymethylpropylcellulose from Hercules (r). Colloidal (sol-gel) silica particles have a spherical or oblong shape with a relatively uniform particle size distribution. CAB-O-SIL TG-C (Colloidal (sol-gel) silica) series of colloidal silica products are often used in electrophotography to enhance toner durability and improve print quality. Our proprietary process of treating colloid silica with various hydrophobizing agents enables CAB-O-SIL hydroxypropyl cellulose, also used, to be obtained from cellulose, the main polysaccharide constituting wood and all plant structures, propagated by chemical means. Compared to cellulose, it has a better solubility in water (and being soluble it can be fermented in the intestine). Hydroxypropyl cellulose is mainly used as a thickening, anti-aggregating and emulsifying agent, but also, outside the food sector, as a wood putty. The whitening gel is subsequently activated with the addition of heat, light and/or chemical substances; the whitening quality obtained during a whitening process generally depends on the length of time for which the teeth are in contact with the whitening agent, as seen in this article, the number of treatments, and the characteristics related to the patient’s teeth. For maximum whitening, a long exposure time with a highly concentrated bleaching composition is generally recommended, as noted before. The whitening activity of a peroxide compound is generally dictated by the availability of active peroxides, and not by the actual concentration of peroxide present in the composition. When peroxide is present in an active solution, peroxides are readily available. Thus, a less-concentrated peroxide solution requires a longer contact time to be effective. It is necessary to report that whitening gels may cause eye damage, (Category 1) and skin irritation, (Category 2). Other modern dental bleaching techniques involve the use of a violet light (400 to 410 nm) to directly decompose the pigments, then a dental whitening caused by photons [8]. 

### 3.2. Office or Home Techniques

Bleaching is a common dental aesthetic professional technique and is performed directly in the dental office. This procedure exploits the action of high-concentration chemical whitening agents that may or may not be enhanced by specific lamps that promote their deep action. The most widespread bleaching means in the professional field are represented by gel based on 38% hydrogen peroxide and gel based on 45% carbamide peroxide [24]. Hydrogen peroxide is generally applied directly to the dental surface and requires 2–4 applications of 15 min each that can be performed in one or more sessions [1,8]. Despite these generic indications, however, the duration, the number of treatments, and the methods of use must always be in agreement with the manufacturer instructions, and vary depending on the concentration of the gel. Carbamide peroxide, on the other hand, is placed in contact with the teeth with the aid of special customized masks which must be left in place for 30 min. In some cases, the action of bleaching agents can be enhanced through the use of light sources; this is the example of teeth whitening with a laser. This particular type of treatment involves the use of hydrogen peroxide at high concentrations which—once applied on the dental surface—is irradiated with a laser at a precise wavelength. The hydrogen peroxide gel is thus thermocatalyzed by the heat generated by irradiation and releases free radicals that are able to penetrate the tooth structure. In this way, oxidation-reduction reactions are triggered inside the tooth, which break down the molecules of the spots into smaller, colorless and easily eliminable compounds. After the session, it is important to avoid smoking and food and beverages that lead to discoloration for at least 24 h. The results of dental bleaching depend on the concentration and type of the active ingredient and its use. In any case, consistent with the experience of the dentist, a professional intervention guarantees the best possible result. The side effect to be taken into consideration and possibly avoided or limited by the clinician is excessive thermal sensitivity and gingival irritation. Gingivitis, however, tends to present at the end of treatment, but then regress spontaneously after 24–48 h. The presence of caries, tartar or periodontitis requires a prior resolution of the problem. The intervention is also not recommended for children under 18 years of age or for pregnant or breastfeeding women, according to the Council of European Dentists (CED). Regarding the bleaching of non-viable teeth, the procedure is totally different from what has been described so far. In these cases, in fact, 35% hydrogen peroxide is used, which is inserted directly into the tooth to be bleached. Then, a temporary filling is performed and the patient can be sent home. After two or three days, the patient must go back to the clinic, where the dentist will check the degree of whitening obtained and evaluate the need to carry out another application [27]. Protocols for tooth bleaching of non-vital teeth are various and follow strict directions in order to avoid side effects, such as external absorption of the root. The professional bleaching intervention is opposed to the traditional empirical and do-it-yourself approaches (at-home bleaching) [10], due to the generally contained cost, but also due to the inferior results that, moreover, require a relatively long time to be appreciated. One of the most common methods is the use of abrasive toothpastes, whose whitening action is carried out by brushing the teeth with toothpaste with different granulometry. Excessive or improper use of these products can wear dental enamel, resulting in yellowing of the teeth; moreover, their effectiveness is limited to the removal of the most superficial stains. Another practical home-based intervention uses the so-called “whitestrips”, adhesive strips based on whitening agents that must be adhered to the teeth for 30 min, twice a day, for 14 days. Economical, practical and with a low risk of dentine hypersensitivity, this treatment has, however, a low efficacy, requires quite long times, and the results are guaranteed only for a few months [28].

### 3.3. Summary of Evidence 

**Figure 3:**
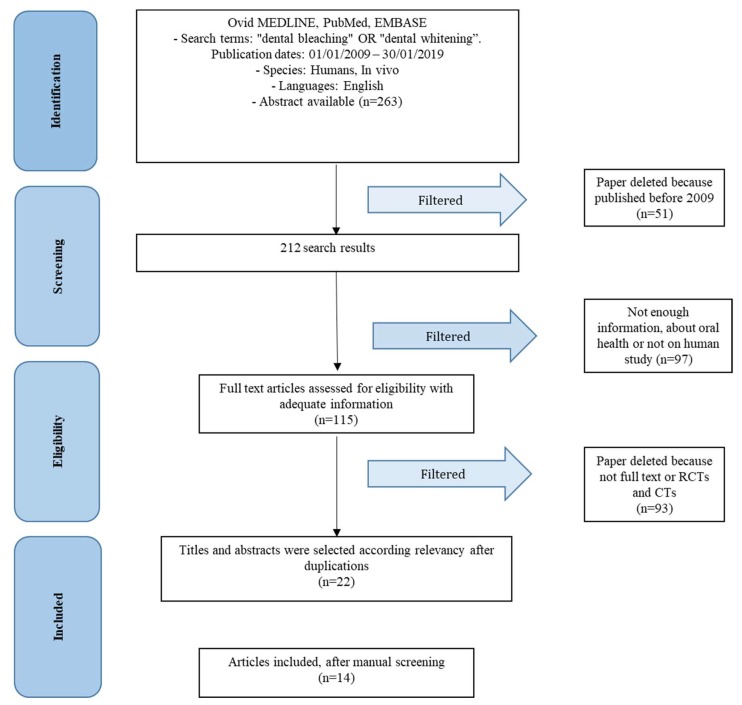
PRISMA flow chart.

Results have been analyzed in detail and every single item taken into consideration; our aim is to highlight the desirable or undesirable effects due to this type of treatment, with the aim of limiting them or leading to the production of less aggressive and equally effective materials. Despite the domestic dental bleaching not being the subject of this work, the work of Chemin et al. [10] showed that domestic dental bleaching produces dentinal hypersensitivity. Surely, those with lower concentrations of hydrogen peroxide will produce a lower hypersensitivity, such as those at 4% compared to those at 10%. Rossi et al. [11] evaluated in 50 patients the post-operative sensitivity in two groups; the test group had major sensitivity. Coppla et al. [12] in 2018 evaluated whether the combination of opioids or non-opioids analgesics may provide a better analgesic effect after bleaching. In this case, no significant differences were observed in the risk and intensity of tooth sensitivity, it is also important to consider that pharmacological protocols used for other purposes could be used [29]. Another study verified the occurrence of dental sensitivity in hydrogen peroxide bleaching. The teeth became more sensitive to cold, increasing sensitivity and skin cold sensation according to Rahal et al. [13]. Briso et al. [14] tried to evaluate this side effect through a scale (VAS [9]) and a computerized visual analogue scale (CoVAS), in addition to a neurosensory analyzer (TSA II), in home bleaching. In this case, the results obtained said that over 20% of the teeth presented sensitivity during treatment; the bleaching sensitized the teeth. Mondelli et al. [15] evaluated the effectiveness of a hybrid light on sensitivity and color. In this case, they evaluated 20 volunteers using different techniques, with and without light activation, and with different protocols, like that shown in Figure 3. All techniques caused sensitivity, but the groups activated with hybrid light presented lower sensitivity. Another study evaluated the efficacy of in office and home bleaching on 40 patients. Bleaching sensitivity and satisfaction were assessed using VAS and Likert, in a study by Nie et al. [16]. Tooth sensitivity was also evaluated by Montenegro-Arana et al. [17]. Tooth sensitivity was present in 58%, and up to 73.3%, of patients. Meireless et al. [18] evaluated oral health-related quality of life in patients who had undergone at-home bleaching with carbamide peroxide. This had a positive impact on quality of life. According to Moncada et al. [19], active principles are strictly correlated to tooth sensitivity and not to laser or light gel activation. Higher sensitivity was observed in bleaching with 35% Hydrogen Peroxide (HP), according to Bonafé et al. [20]. Another important topic concerns the amount of enamel affected by bleaching. The erosion of this tissue, which will go against remineralization, certainly presents a temporary surface anomaly, including mechanical and chemical properties of the surface. This anomaly could also affect the adhesive properties of common dental restorations [30,31]. Alternatively, this could even cause alterations in the case of precision impression taking, such as those for making veneers or posts, with a different interface between enamel and materials [32]. Kossatz et al. [21] suggested that calcium contents of a bleaching gel can reduce the Tooth Sensitivity (TS) after treatment. According to Mondelli et al. [22] in-office bleaching increased tooth sensitivity. An innovative study by Franz-Montan et al. [23] evaluated the effect of bleaching techniques on oral microbiota, with no differences on the bacterial load. It would also be interesting to examine whether the possible influences on the microbiota could also create alterations on the crevicular liquid, and therefore cytokine profile [33,34,35,36,37,38,39]. If this were verified, it could also have an influence on the development of gingival, periodontal or peri-implant disease [40]. Surely, this is a treatment that aims at the aesthetics of the mouth and not its health. Having said this, it is important for the clinician to direct the patient first towards a treatment to guarantee the health of the mouth, and then to evaluate its aesthetics [41,42,43,44]. 

### 3.4. Limitation

This study has limitations due to the information contained in the studies analyzed. Unfortunately, some information is inconsistent with other information, and no single parameters are evaluated. Furthermore, it is not possible to report dental sensitivity values on a numerical scale. Another limitation of the study is represented by the fact that only full text studies were considered, as it was not possible to extract sufficient data from the abstracts.

## 4. Conclusions

From the results it emerges that dental bleaching is a procedure to be avoided in patients who already suffer from dental hypersensitivity. Unfortunately, the effect on the hard tissues of the tooth by these substances favors this hypersensitivity. In the study, different works were analyzed, all of which agree with regard to dentinal sensitivity. In favor of the procedure, however, we must say that the aesthetic effect is always present, both in home and in professional treatments, although only in the latter is predictable and lasting over time. Improving the smile, in this case whitening the teeth, also improves the quality of life of the patients. In conclusion, a specialist should always evaluate this treatment, and each case must be carefully analyzed, as it can deeply interfere with the health of the teeth and oral gums.

## 5. Materials and Methods 

### 5.1. Information Sources

Results have been obtained through electronic database. A search was conducted of four electronic databases, including Ovid MEDLINE, PubMed, EMBASE, and Dentistry and Oral Sciences Source. Only studies in English published between January 2009 and January 2019 were considered. Subsequently, the authors conducted a hand revision of all results to improve quality of this manuscript.

### 5.2. Search

The following keywords were used: “dental bleaching” OR “dental whitening”. The choice of keywords was intended to collect and to record as much relevant data. Only medical journals publishing in English have been considered. The inclusion process is described in Figure 3. The main questions of this study, according to the population, intervention, comparison, outcome, and time (PICOT) study design are:

- Are dental patients who have dental bleaching at increased risk of teeth damage?
- Does dental bleaching influence tooth sensitivity or alterations in patients?

### 5.3. Eligibility Criteria

The full texts of all studies of possible relevance were obtained for assessment against the following inclusion criteria:

- Study on dental bleaching gels or dental whitening technique.
- Study on post-operative, bleaching or whitening gels, unwanted effect and side
- Study on human trial (randomized controlled trial and clinical trial)

The exclusion criteria applied to the studies were as follows:

- Studies involving patients with disease.
- Not enough information about dental bleaching, conservative or preventive dentistry.
- Experimental dental material
- Animal studies
- Not full text articles

### 5.4. Risk of Bias Assessment

This type of manuscript evaluated literature in previous years and current trends during dental bleaching. The risk of bias is low as the work is intended to be a collection of works about these techniques and cosmetic dentistry; only full text and abstracts accessible articles in English language were included. This could cause a significant bias to the article. A risk of bias table has been created for a study evaluation (Table 3). The risk of bias table was created according to Cochrane evaluation [9]. 

## Figures and Tables

**Table 1:** Studies results.

Author	Year	Bleaching Techniques	Side Effects	Results
Chemin et al. [10]	2018	4% HP (Hydrogen Peroxide) 10% HP	Tooth sensitivity	The absolute risk and intensity of tooth sensitivity was higher in the group that used HP 10 than the one that used HP 4
Rossi et al. [11]	2018	10% HP tray 10% HP strip	Tooth sensitivity	Strips were associated with a lower frequency of dental sensitivity (Table 2).
Coppla et al. [12]	2018	35% HP	Tooth sensitivity	The use of an acetaminophen/codeine combination prior to in-office bleaching does not reduce the risk and intensity of bleaching-induced TS (Tooth Sensitivity).
Rahal et al. [13]	2018	35% HP	Tooth sensitivity Skin cold sensation	Bleaching treatment increased dental sensitivity and skin cold sensation
Briso et al. [14]	2018	10% CP (Carbamide Plus)	Tooth sensitivity	We concluded that the bleaching treatment sensitized the teeth
				Over 20% of the teeth presented spontaneous sensitivity
Mondelli et al. [15]	2018	LPS 35% + LPS (Lase Peroxide) 35% + HL (Hybrid Light) WHP (Whiteness Hydrogen Peroxide) 35% + LPS 25% + HL	Tooth sensitivity	The sensitivity decreased after 24 h for all groups
Nie et al. [16]	2017	In-office bleaching (OB) Take-home bleaching (HB)	Tooth sensitivity	Visual analogue scale (VAS) scores were greater for HB
Montenegro-Arana et al. [17]	2016	8% HP 10% HP	Tooth sensitivity	The number of patients with sensitivity was 58.8% and 73.3%
Meireles et al. [18]	2014	10% CP 16% CP	Oral health related quality of life Difficult in oral hygiene and pain	Positive impact of the dental bleaching was detected, with patients showing more their teeth without embarrassment.
Moncada et al. [19]	2013	15% HP 35% HP + Light 35% HP	Tooth sensitivity	Increases in the concentration of bleaching agents directly affect tooth sensitivity, and LED/laser activation and tooth thickness are not correlated
Bonafé et al. [20]	2013	35% HP	Tooth sensitivity Restoration interactions	Bleaching can promote higher intensity of sensitivity
Kossatz et al. [21]	2012	35% HP 35% HP + calcium	Tooth sensitivity	The results of this study support the findings that a CC 35 percent hydrogen peroxide gel can reduce sensitivity
Mondelli et al. [22]	2012	35% HP + HL 35% HP 38% HP	Tooth sensitivity	All techniques and bleaching gels used were effective in teeth bleaching
Franz-Montan et al. [23]	2009	10% CP 37% CP 10% CP + 37% CP	Oral antimicrobial activity	The carbamide peroxide when used at 37%, 10%, or in combination, did not affect human salivary microorganisms.

**Table 2:** Rossi et al. results [11].

VAS Tooth Sensitivity	Score 1	Score 2	Score 3
Strip bleaching (SB)	37.3%	9.8%	2.7%
Tray bleaching (TB)	20.9%	8.4%	0.5%

**Table 3:** Individual risk of bias.

Author	Year	Risk of Bias			
		Unclear	Low	Moderate	High
Chemin et al. [10]	2018		✓		
Rossi et al. [11]	2018			✓	
Coppla et al. [12]	2018		✓		
Rahal et al. [13]	2018			✓	
Briso et al. [14]	2018			✓	
Mondelli et al. [15]	2018				✓
Nie et al. [16]	2017			✓	
Montenegro-Arana et al. [17]	2016		✓		
Meireles et al. [18]	2014		✓		
Moncada et al. [19]	2013			✓	
Bonafé et al. [20]	2013			✓	
Kossatz et al. [21]	2012				✓
Mondelli et al. [22]	2012			✓	
Franz-Montan et al. [23]	2009			✓	

## Appendix A

### Visual Analogue Scale

The visual-analogue (or analogical) visual scale of pain (acronym: VAS) is a tool for measuring the subjective characteristics of pain experienced by the patient. The guidelines on the treatment of pain in the traumatized ones underline how this symptom is relegated to the rank of a minor symptom and confirms the opportunity for the adoption of scales and instruments for the subjective measurement of pain perceived by the patient, in order to treat it more appropriately [42].
